# (*E*)-1,2-Diphenyl­ethenyl methane­sulfonate

**DOI:** 10.1107/S160053681001281X

**Published:** 2010-04-24

**Authors:** Yonghua Feng, Liu Ying, Chen Zhang

**Affiliations:** a Zhejiang Xianju Xianle Pharmaceutical Co. Ltd, People’s Republic of China; b Zhejiang Silver-Elephant Bio-Engineering Co. Ltd, People’s Republic of China; c Department of Medicinal Chemistry, College of Pharmaceutical Science, Zhejiang University, People’s Republic of China

## Abstract

In the title compound, C_15_H_14_O_3_S, the dihedral angle between the two benzene rings is 59.3 (8)°. The crystal structure is stabilized by weak inter­molecular C—H⋯π inter­actions between the benzene rings and the central ethyl­ene bridge, and a weak non-classical C—H⋯O hydrogen bond occurs in the structure.

## Related literature

For general background to the design and synthesis of vinyl sulfonate derivatives, see: Limmert *et al.* (2005 ▶). For related structures, see: Cui *et al.* (2009 ▶). For bond-length data, see: Allen *et al.* (1987 ▶).
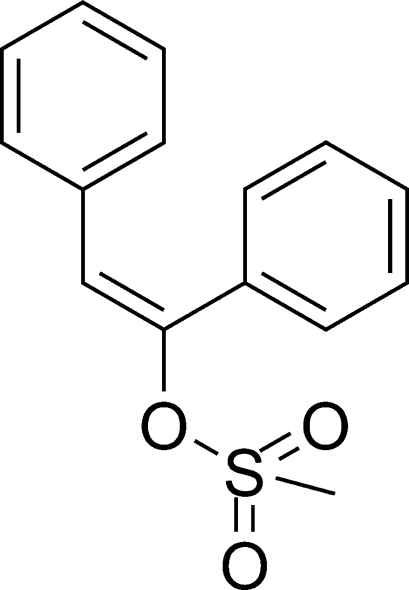


## Experimental

### 

#### Crystal data

C_15_H_14_O_3_S
*M* *_r_* = 274.33
Orthorhombic, 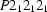

*a* = 8.3789 (3) Å
*b* = 11.1397 (4) Å
*c* = 14.8365 (5) Å
*V* = 1384.82 (8) Å^3^
*Z* = 4
Mo *K*α radiation
μ = 0.23 mm^−1^
*T* = 296 K
0.41 × 0.39 × 0.29 mm

#### Data collection

Rigaku R-AXIS RAPID diffractometer
Absorption correction: multi-scan (*ABSCOR*; Higashi, 1995 ▶) *T*_min_ = 0.887, *T*_max_ = 0.934
13673 measured reflections
3163 independent reflections
2606 reflections with *F* ^2^ > 2.0σ(*F* ^2^)
*R*_int_ = 0.026

#### Refinement

*R*[*F* ^2^ > 2σ(*F* ^2^)] = 0.032
*wR*(*F* ^2^) = 0.092
*S* = 1.00
3163 reflections
174 parameters
H-atom parameters constrained
Δρ_max_ = 0.15 e Å^−3^
Δρ_min_ = −0.20 e Å^−3^
Absolute structure: Flack (1983 ▶), 1341 Friedel Pairs
Flack parameter: −0.03 (7)

### 

Data collection: *PROCESS-AUTO* (Rigaku, 2006 ▶); cell refinement: *PROCESS-AUTO*; data reduction: *CrystalStructure* (Rigaku, 2007 ▶); program(s) used to solve structure: *SIR97* (Altomare *et al.*, 1999 ▶); program(s) used to refine structure: *SHELXL97* (Sheldrick, 2008 ▶); molecular graphics: *ORTEP-3 for Windows* (Farrugia, 1997 ▶); software used to prepare material for publication: *CrystalStructure* (Rigaku Americas, 2007 ▶).

## Supplementary Material

Crystal structure: contains datablocks global, I. DOI: 10.1107/S160053681001281X/bq2190sup1.cif

Structure factors: contains datablocks I. DOI: 10.1107/S160053681001281X/bq2190Isup2.hkl

Additional supplementary materials:  crystallographic information; 3D view; checkCIF report

## Figures and Tables

**Table 1:** Hydrogen-bond geometry (Å, °)

*D*—H⋯*A*	*D*—H	H⋯*A*	*D*⋯*A*	*D*—H⋯*A*
C8—H8⋯O2^i^	0.93	2.53	3.376 (2)	152
C15—H152⋯*Cg*1^ii^	0.96	2.68	3.514 (1)	145

Symmetry codes: (i) 
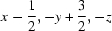
; (ii) 
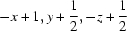
. *Cg*1 is the centroid of the C9–C14 ring.

**Fig. 1.**
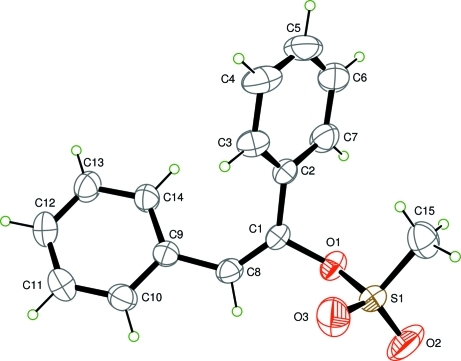
The molecular structure of (I) with atom labels showing the 50% probability displacement ellipsoids.

**Fig. 2.**
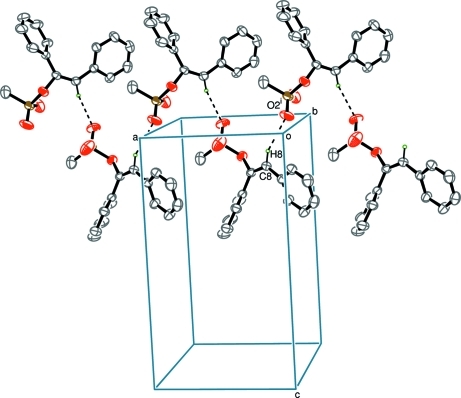
The packing of (I) viewed down the a-axis. Hydrogen bonds are shown as dashed lines. Symmetry code: (i) -0.5+x, 1.5-y, -z.

## supplementary crystallographic information

### Comment

Vinyl sulfonates, important building blocks in organic synthesis, especially as electrophiles for cross-coupling chemistry, have received much attention in recent years. These kinds of compounds are not generally stable. The title compound (I) seems to be stable by weak intermolecular interactions (Figure 2) between the benzene rings and central ethylene bridge, and also weak non-classical H bond occurs in the structure (Table 1). In (I), all bond lengths and angles are normal (Allen *et al.*, 1987), and the dihedral angle between the two benzene rings is 59.3 (8)° (Figure 1).

### Experimental

1,2-diphenylethyne and methanesulfonic acid in the presence of a catalytic amount of (Ph3P)AuNO_3_ (5 mol%) and phthalimide (10 mol%) in dichloroethane was stirred for 8 h at 373 K. It was quenched with saturated solution of NaHCO_3_ and then extracted with ethyl acetate (3 x 10 ml). The organic layer was washed with brine, dried over Na_2_SO_4_ and concentrated in vacuo. The residue was purified by flash chromatography to give the pure product. It proceeds efficiently to form the adduct in 73% yield.

### Refinement

All H atoms were placed in calculated positions, with C—H distances in the range 0.93-0.98 and included in the final cycles of refinement in the riding-model approximation, with Uiso(H) = k1.2Ueq(C).

### Crystal data

**Table:**

C_15_H_14_O_3_S	*F*(000) = 576.00
*M**_r_* = 274.33	*D*_x_ = 1.316 Mg m^−^^3^
Orthorhombic, *P*2_1_2_1_2_1_	Mo *K*α radiation, λ = 0.71075 Å
Hall symbol: P 2ac 2ab	Cell parameters from 11138 reflections
*a* = 8.3789 (3) Å	θ = 3.0–27.4°
*b* = 11.1397 (4) Å	µ = 0.23 mm^−^^1^
*c* = 14.8365 (5) Å	*T* = 296 K
*V* = 1384.82 (8) Å^3^	Chunk, colorless
*Z* = 4	0.41 × 0.39 × 0.29 mm

### Data collection

**Table:**

Rigaku R-AXIS RAPID diffractometer	2606 reflections with *F*^2^ > 2.0σ(*F*^2^)
Detector resolution: 10.00 pixels mm^-1^	*R*_int_ = 0.026
ω scans	θ_max_ = 27.4°
Absorption correction: multi-scan (*ABSCOR*; Higashi, 1995)	*h* = −10→10
*T*_min_ = 0.887, *T*_max_ = 0.934	*k* = −13→14
13673 measured reflections	*l* = −19→18
3163 independent reflections	

### Refinement

**Table:**

Refinement on *F*^2^	(Δ/σ)_max_ < 0.001
*R*[*F*^2^ > 2σ(*F*^2^)] = 0.032	Δρ_max_ = 0.15 e Å^−^^3^
*wR*(*F*^2^) = 0.092	Δρ_min_ = −0.20 e Å^−^^3^
*S* = 1.00	Extinction correction: *SHELXL97* (Sheldrick, 2008)
3163 reflections	Extinction coefficient: 0.0173 (19)
174 parameters	Absolute structure: Flack (1983), 1341 Friedel Pairs
H-atom parameters constrained	Flack parameter: −0.03 (7)
*w* = 1/[σ^2^(*F*_o_^2^) + (0.0507*P*)^2^ + 0.1656*P*] where *P* = (*F*_o_^2^ + 2*F*_c_^2^)/3	

### Special details

**Table:**

Refinement. Refinement using all reflections. The weighted *R*-factor (wR) and goodness of fit (*S*) are based on *F*^2^. *R*-factor (gt) are based on *F*. The threshold expression of *F*^2^ > 2.0 σ(*F*^2^) is used only for calculating *R*-factor (gt).

### Fractional atomic coordinates and isotropic or equivalent isotropic displacement parameters (Å^2^)

**Table:**

	*x*	*y*	*z*	*U*_iso_*/*U*_eq_	
S1	0.61979 (6)	0.70614 (5)	0.09128 (3)	0.05358 (15)	
O1	0.47962 (16)	0.75814 (11)	0.15261 (8)	0.0510 (3)	
O2	0.6188 (2)	0.78154 (19)	0.01488 (11)	0.1006 (6)	
O3	0.6001 (2)	0.58046 (14)	0.08152 (12)	0.0874 (5)	
C1	0.3992 (2)	0.68265 (13)	0.21579 (10)	0.0413 (3)	
C2	0.4741 (2)	0.68403 (14)	0.30602 (11)	0.0426 (3)	
C3	0.4984 (2)	0.57768 (18)	0.35269 (12)	0.0528 (4)	
C4	0.5742 (2)	0.5788 (2)	0.43570 (13)	0.0720 (6)	
C5	0.6285 (3)	0.6847 (2)	0.47133 (13)	0.0848 (7)	
C6	0.6049 (3)	0.7911 (2)	0.42591 (13)	0.0842 (7)	
C7	0.5272 (2)	0.7914 (2)	0.34348 (12)	0.0635 (5)	
C8	0.2696 (2)	0.62793 (16)	0.18488 (12)	0.0462 (4)	
C9	0.1462 (2)	0.56029 (14)	0.23397 (11)	0.0429 (3)	
C10	0.0528 (2)	0.47874 (19)	0.18648 (13)	0.0553 (4)	
C11	−0.0714 (2)	0.4185 (2)	0.22810 (16)	0.0649 (5)	
C12	−0.1058 (2)	0.43858 (18)	0.31661 (16)	0.0612 (5)	
C13	−0.0169 (2)	0.51958 (19)	0.36462 (14)	0.0608 (5)	
C14	0.1080 (2)	0.57984 (17)	0.32428 (12)	0.0522 (4)	
C15	0.7899 (2)	0.7341 (2)	0.15510 (18)	0.0714 (6)	
H3	0.4637	0.5054	0.3282	0.063*	
H4	0.5884	0.5075	0.4673	0.086*	
H5	0.6815	0.6847	0.5264	0.102*	
H6	0.6411	0.8628	0.4506	0.101*	
H7	0.5106	0.8634	0.3132	0.076*	
H8	0.2544	0.6331	0.1229	0.055*	
H10	0.0741	0.4645	0.1259	0.066*	
H11	−0.1320	0.3637	0.1954	0.078*	
H12	−0.1891	0.3975	0.3443	0.073*	
H13	−0.0409	0.5341	0.4248	0.073*	
H14	0.1677	0.6343	0.3578	0.063*	
H151	0.8829	0.7131	0.1208	0.086*	
H152	0.7940	0.8178	0.1705	0.086*	
H153	0.7865	0.6870	0.2092	0.086*	

### Atomic displacement parameters (Å^2^)

**Table:**

	*U*^11^	*U*^22^	*U*^33^	*U*^12^	*U*^13^	*U*^23^
S1	0.0561 (2)	0.0659 (2)	0.0387 (2)	−0.0098 (2)	0.0083 (2)	−0.0016 (2)
O1	0.0526 (7)	0.0517 (6)	0.0486 (6)	0.0011 (5)	0.0082 (5)	0.0140 (5)
O2	0.0944 (13)	0.1520 (18)	0.0553 (8)	0.0003 (14)	0.0209 (9)	0.0421 (10)
O3	0.0931 (12)	0.0749 (10)	0.0941 (11)	−0.0237 (9)	0.0330 (11)	−0.0417 (9)
C1	0.0450 (9)	0.0403 (8)	0.0387 (7)	0.0011 (7)	0.0059 (7)	0.0078 (6)
C2	0.0409 (8)	0.0484 (9)	0.0384 (7)	−0.0008 (7)	0.0074 (6)	−0.0006 (7)
C3	0.0595 (11)	0.0550 (10)	0.0437 (9)	0.0102 (9)	−0.0020 (8)	0.0018 (8)
C4	0.0755 (15)	0.0970 (17)	0.0437 (10)	0.0231 (14)	−0.0026 (9)	0.0089 (11)
C5	0.0799 (16)	0.136 (2)	0.0381 (9)	−0.0057 (19)	−0.0035 (11)	−0.0126 (13)
C6	0.1004 (19)	0.1041 (18)	0.0483 (11)	−0.0390 (17)	0.0141 (12)	−0.0247 (12)
C7	0.0851 (15)	0.0571 (10)	0.0484 (9)	−0.0150 (11)	0.0131 (10)	−0.0055 (9)
C8	0.0468 (9)	0.0533 (10)	0.0384 (8)	0.0025 (8)	−0.0015 (7)	0.0060 (7)
C9	0.0408 (9)	0.0441 (8)	0.0438 (8)	0.0030 (7)	−0.0029 (7)	0.0030 (7)
C10	0.0524 (10)	0.0632 (11)	0.0504 (9)	−0.0025 (9)	−0.0063 (9)	−0.0050 (9)
C11	0.0552 (12)	0.0616 (12)	0.0777 (14)	−0.0128 (10)	−0.0067 (10)	−0.0075 (11)
C12	0.0477 (10)	0.0565 (10)	0.0795 (13)	−0.0095 (9)	0.0092 (10)	0.0034 (10)
C13	0.0573 (12)	0.0659 (12)	0.0591 (11)	−0.0072 (10)	0.0161 (10)	−0.0025 (10)
C14	0.0493 (9)	0.0560 (10)	0.0511 (9)	−0.0091 (9)	0.0067 (9)	−0.0061 (8)
C15	0.0518 (11)	0.0768 (15)	0.0857 (16)	−0.0016 (11)	0.0009 (10)	−0.0069 (13)

### Geometric parameters (Å, °)

**Table:**

S1—O1	1.5946 (13)	C11—C12	1.363 (3)
S1—O2	1.4108 (18)	C12—C13	1.370 (3)
S1—O3	1.4172 (17)	C13—C14	1.380 (2)
S1—C15	1.739 (2)	C3—H3	0.930
O1—C1	1.428 (2)	C4—H4	0.930
C1—C2	1.478 (2)	C5—H5	0.930
C1—C8	1.328 (2)	C6—H6	0.930
C2—C3	1.387 (2)	C7—H7	0.930
C2—C7	1.392 (2)	C8—H8	0.930
C3—C4	1.386 (2)	C10—H10	0.930
C4—C5	1.370 (4)	C11—H11	0.930
C5—C6	1.378 (4)	C12—H12	0.930
C6—C7	1.385 (3)	C13—H13	0.930
C8—C9	1.472 (2)	C14—H14	0.930
C9—C10	1.391 (2)	C15—H151	0.960
C9—C14	1.395 (2)	C15—H152	0.960
C10—C11	1.384 (3)	C15—H153	0.960
			
O1—S1—O2	103.76 (10)	C4—C3—H3	119.9
O1—S1—O3	109.35 (9)	C3—C4—H4	119.9
O1—S1—C15	103.16 (9)	C5—C4—H4	119.9
O2—S1—O3	120.36 (11)	C4—C5—H5	119.9
O2—S1—C15	109.61 (12)	C6—C5—H5	119.9
O3—S1—C15	109.16 (11)	C5—C6—H6	119.9
S1—O1—C1	120.53 (10)	C7—C6—H6	119.9
O1—C1—C2	112.84 (13)	C2—C7—H7	120.0
O1—C1—C8	115.45 (14)	C6—C7—H7	120.0
C2—C1—C8	131.66 (15)	C1—C8—H8	115.1
C1—C2—C3	120.36 (15)	C9—C8—H8	115.1
C1—C2—C7	120.41 (15)	C9—C10—H10	119.5
C3—C2—C7	119.18 (16)	C11—C10—H10	119.5
C2—C3—C4	120.20 (19)	C10—C11—H11	119.7
C3—C4—C5	120.2 (2)	C12—C11—H11	119.7
C4—C5—C6	120.3 (2)	C11—C12—H12	120.2
C5—C6—C7	120.1 (2)	C13—C12—H12	120.2
C2—C7—C6	120.0 (2)	C12—C13—H13	119.8
C1—C8—C9	129.70 (16)	C14—C13—H13	119.8
C8—C9—C10	118.61 (15)	C9—C14—H14	119.5
C8—C9—C14	123.81 (15)	C13—C14—H14	119.5
C10—C9—C14	117.37 (16)	S1—C15—H151	109.5
C9—C10—C11	120.93 (19)	S1—C15—H152	109.5
C10—C11—C12	120.6 (2)	S1—C15—H153	109.5
C11—C12—C13	119.6 (2)	H151—C15—H152	109.5
C12—C13—C14	120.5 (2)	H151—C15—H153	109.5
C9—C14—C13	120.97 (18)	H152—C15—H153	109.5
C2—C3—H3	119.9		
			
O2—S1—O1—C1	154.37 (13)	C2—C3—C4—C5	−1.3 (3)
O3—S1—O1—C1	24.77 (15)	C3—C4—C5—C6	1.5 (3)
C15—S1—O1—C1	−91.29 (14)	C4—C5—C6—C7	−0.6 (4)
S1—O1—C1—C2	90.79 (14)	C5—C6—C7—C2	−0.5 (3)
S1—O1—C1—C8	−91.52 (17)	C1—C8—C9—C10	−158.77 (19)
O1—C1—C2—C3	−135.28 (16)	C1—C8—C9—C14	26.6 (2)
O1—C1—C2—C7	41.9 (2)	C8—C9—C10—C11	−175.86 (18)
O1—C1—C8—C9	−169.44 (16)	C8—C9—C14—C13	175.14 (18)
C2—C1—C8—C9	7.7 (3)	C10—C9—C14—C13	0.5 (2)
C8—C1—C2—C3	47.5 (2)	C14—C9—C10—C11	−0.9 (2)
C8—C1—C2—C7	−135.3 (2)	C9—C10—C11—C12	0.6 (3)
C1—C2—C3—C4	177.46 (18)	C10—C11—C12—C13	0.3 (3)
C1—C2—C7—C6	−176.5 (2)	C11—C12—C13—C14	−0.7 (3)
C3—C2—C7—C6	0.7 (3)	C12—C13—C14—C9	0.3 (3)
C7—C2—C3—C4	0.2 (2)		

### Hydrogen-bond geometry (Å, °)

**Table:**

*D*—H···*A*	*D*—H	H···*A*	*D*···*A*	*D*—H···*A*
C8—H8···O2^i^	0.93	2.53	3.376 (2)	152
C15—H152···Cg1^ii^	0.96	2.68	3.514 (1)	145

Symmetry codes: (i) *x*−1/2, −*y*+3/2, −*z*; (ii) −*x*+1, *y*+1/2, −*z*+1/2.
